# White matter microstructural and morphometric alterations in autism: implications for intellectual capabilities

**DOI:** 10.1186/s13229-022-00499-1

**Published:** 2022-05-18

**Authors:** Chun-Hung Yeh, Rung-Yu Tseng, Hsing-Chang Ni, Luca Cocchi, Jung-Chi Chang, Mei-Yun Hsu, En-Nien Tu, Yu-Yu Wu, Tai-Li Chou, Susan Shur-Fen Gau, Hsiang-Yuan Lin

**Affiliations:** 1 Institute for Radiological Research, Chang Gung University, No. 259, Wenhua 1st Road, Guishan District, 333 Taoyuan City, Taiwan; 2 Department of Psychiatry, Chang Gung Memorial Hospital at Linkou, Taoyuan, Taiwan; 3 Clinical Brain Networks Group, QIMR Berghofer Medical Research Institute, Brisbane, QLD Australia; 4 Department of Psychiatry, National Taiwan University Hospital and College of Medicine, Taipei, Taiwan; 5 YuNing Clinic, Taipei, Taiwan; 6 Department of Psychiatry, University of Oxford, Oxford, UK; 7 Department of Psychiatry, Keelung Chang Gung Memorial Hospital, Keelung, Taiwan; 8 Department of Psychology, National Taiwan University, Taipei, Taiwan; 9 Azrieli Adult Neurodevelopmental Centre, Campbell Family Mental Health Research Institute, and Adult Neurodevelopmental and Geriatric Psychiatry Division, Centre for Addiction and Mental Health, 1025 Queen St W – 3314, Toronto, ON M6J 1H4 Canada; 10 Department of Psychiatry and Institute of Medical Science, Temerty Faculty of Medicine, University of Toronto, Toronto, ON Canada

**Keywords:** Autism spectrum disorder, Fixel-based analysis, Intellectual disabilities, Minimally verbal status, Cerebellum, Diffusion MRI

## Abstract

**Background:**

Neuroimage literature of autism spectrum disorder (ASD) has a moderate-to-high risk of bias, partially because those combined with intellectual impairment (II) and/or minimally verbal (MV) status are generally ignored. We aimed to provide more comprehensive insights into white matter alterations of ASD, inclusive of individuals with II (ASD-II-Only) or MV expression (ASD-MV).

**Methods:**

Sixty-five participants with ASD (ASD-Whole; 16.6 ± 5.9 years; comprising 34 intellectually able youth, ASD-IA, and 31 intellectually impaired youth, ASD-II, including 24 ASD-II-Only plus 7 ASD-MV) and 38 demographic-matched typically developing controls (TDC; 17.3 ± 5.6 years) were scanned in accelerated diffusion-weighted MRI. Fixel-based analysis was undertaken to investigate the categorical differences in fiber density (FD), fiber cross section (FC), and a combined index (FDC), and brain symptom/cognition associations.

**Results:**

ASD-Whole had reduced FD in the anterior and posterior corpus callosum and left cerebellum Crus I, and smaller FDC in right cerebellum Crus II, compared to TDC. ASD-IA, relative to TDC, had no significant discrepancies, while ASD-II showed almost identical alterations to those from ASD-Whole vs. TDC. ASD-II-Only had greater FD/FDC in the isthmus splenium of callosum than ASD-MV. Autistic severity negatively correlated with FC in right Crus I. Nonverbal full-scale IQ positively correlated with FC/FDC in cerebellum VI. FD/FDC of the right dorsolateral prefrontal cortex showed a diagnosis-by-executive function interaction.

**Limitations:**

We could not preclude the potential effects of age and sex from the ASD cohort, although statistical tests suggested that these factors were not influential. Our results could be confounded by variable psychiatric comorbidities and psychotropic medication uses in our ASD participants recruited from outpatient clinics, which is nevertheless closer to a real-world presentation of ASD. The outcomes related to ASD-MV were considered preliminaries due to the small sample size within this subgroup. Finally, our study design did not include intellectual impairment-only participants without ASD to disentangle the mixture of autistic and intellectual symptoms.

**Conclusions:**

ASD-associated white matter alterations appear driven by individuals with II and potentially further by MV. Results suggest that changes in the corpus callosum and cerebellum are key for psychopathology and cognition associated with ASD. Our work highlights an essential to include understudied subpopulations on the spectrum in research.

**Supplementary Information:**

The online version contains supplementary material available at 10.1186/s13229-022-00499-1.

## Introduction

Autism spectrum disorder (ASD) is a neurodevelopmental condition, characterized by struggling in socio-communication and the restricted/repetitive behaviors and interests [1]. ASD is associated with categorically and dimensionally neurodevelopmental alterations in brain structures and function, contributing to suboptimal information processing underpinning social communication, sensorimotor integration, and executive control processes [2, 3].

Supporting this observation and extending this understanding to the level of brain connections, studies using diffusion magnetic resonance imaging (dMRI) showed that altered white matter (WM) microstructural properties associated with ASD exist in WM tracts interconnecting brain regions/systems involved in social processing (i.e., the default-mode network approximately corresponding to the “social brain”) and executive control (i.e., the frontoparietal network) [4–7]. For example, increased mean diffusivity (MD) or decreased fractional anisotropy (FA) in the corpus callosum (CC), uncinate fasciculus, superior longitudinal fasciculus, and frontal and temporal thalamic projections have been reported in people with ASD, compared to typically developing control (TDC). However, high variability of findings is commonly noted across studies, partially because of limitations and factors that can affect dMRI results. Specifically, voxel-based analysis (VBA) and tractography of dMRI popularly involve the analysis based on the diffusion tensor model, i.e., DTI [8]. But this model fails to resolve the multiple fiber orientations in regions containing crossing fibers, which exists in most WM voxels [9]. Thus, advanced dMRI models to address this methodological challenge are crucial to advance understanding of the WM pathology associated with ASD.

The state-of-the-art fixel-based analysis, or FBA [10], provides the fiber tract-specific analysis to estimate the quantitative metrics associated with a single fiber population within a voxel (called “fixel”), as opposed to analyses of voxel-averaged metrics. FBA has been shown to be more sensitive and interpretable than voxel-wise methods [11, 12], in terms of better reflection of the local microscopic fiber densities. It also accounts for macroscopic morphometric alterations of fiber bundles such as the overall size in the transverse section, known as fixel-based morphometry [12]. The quantitative measures of FBA can be derived for each identifiable fiber bundle population even given multiple crossing fibers within a voxel. Recently, the FBA has been applied to dMRI studies on ASD [13, 14], showing promising but mixed results. Specifically, Dimond et al*.* [13] reported that able youth with ASD (21 males and 4 females; age = 16.8 ± 2.26 years) have lower microstructural density in the right uncinate and arcuate fasciculi alongside CC-splenium whose fixel fiber density (FD; defined in Methods) is associated with social impairment. Kirkovski et al*.* [14] nevertheless only observed altered micro-/macrostructure in the posterior midbody of CC in able female adults with ASD (12 males and 13 females; age = 30.60 ± 9.14 years). This inconsistency may, in part, reflect different sampling protocols of dMRI (e.g., number of gradient directions and *b* values), resulting in variations of diffusion signals [15] across studies. Disregarding spurious findings introduced by in-scanner head motion [16], as well as sampling heterogeneity among individuals with ASD [17], may also contribute to the discrepancy.

In addition to the above methodological considerations, almost all dMRI studies (including both of the preceding FBA studies [13, 14]) focused only on individuals with relatively intact language and cognitive functioning, limiting the representativeness of the autistic cohort. Nonetheless, around 50% of children with ASD have cognitive and intellectual impairments (i.e., IQ scores < 85, representing intellectual disabilities (ID) plus borderline intelligence [18]), and about 30% of children with ASD remain minimally verbal (MV) by reaching their school age [19]. Unfortunately, these “low-functioning” individuals are understudied and often excluded from neuroimaging research. Despite prominent heterogeneity in designs, samples and findings, results from the limited existing studies suggest that people with ASD and ID have alterations of gray matter (GM) and WM morphology in diffuse regions implicated in ASD pathology [20]. Specifically, children with MV ASD showed structural disruptions in language pathways [19, 20]. In the Autism Phenome Project (APP; 85 males and 40 females; age = 24–42 months at timepoint 1, approximately age range = 2.5–7 years across 2–3 timepoints) or Girls with Autism: Imaging of Neurodevelopment (GAIN; 85 males and 42 females; age = 38.8 ± 5.6 months) cohort [21, 22], children with ASD across the spectrum of developmental levels showed a transition from increased (during toddlerhood) to decreased (2 years later) FA in the CC, superior longitudinal fasciculus, cingulum, and internal capsule. The paucity of studies including individuals with co-occurring developmental disabilities [20] do, however, bias the understanding of brain bases of ASD [23].

Using the state-of-the-art dMRI acquisition (four b values with ~ 200 gradient directions), data processing framework (FBA alongside the rigorous diffusion-weighted image (DWI) preprocessing including motion denoising), as well as the extensive psychopathological measures, the current study aimed to provide more comprehensive insights into the structural brain changes underpinning ASD. The current study included individuals with ID and MV, who are generally left out in the current lore. We hypothesized that the FBA could highlight potential differences in ASD that are driven by intellectual challenges. Based on previous work, we expected that intellectually able individuals with ASD would show alterations of WM fibers in the CC [13, 14], uncinate and superior longitudinal fasciculi, and the thalamic radiation. Individuals with ASD plus cognitive impairment (including those with MV expression) would show alterations with broader spatial involvement. Autistic traits, cognitive impairment, and poor adaptive function were in fact expected to map onto fixel pathology in the tract interconnecting social (including the cingulate bundle interconnecting posterior cingulate and medial prefrontal cortex, the anterior CC interconnecting bilateral inferior frontal gyrus alongside mid-posterior CC interconnecting bilateral temporoparietal junction and superior temporal sulcus, the superior longitudinal fasciculus connecting inferior frontal gyrus and temporoparietal junction, and the uncinate fasciculus connecting amygdala and ventromedial prefrontal cortex) and cognitive brain networks (especially the superior longitudinal fasciculus interconnecting dorsolateral prefrontal cortex and inferior parietal lobule).

## Methods

### Procedures and participants

The study was approved by the Research Ethics Committee of National Taiwan University Hospital (#201512238RINC). Participants with ASD (aged 8–30 years) were recruited from the outpatient psychiatric clinic at National Taiwan University Hospital, Chang Gung Memorial Hospital, Linkou, and Yuning Clinic. Age-matched TDC were recruited from neighborhoods with a similar socioeconomic environment to the ASD group. Before implementing the experiment, for those capable of giving consent themselves (i.e., showing capacity to understand the protocol), written informed consent was obtained from each participant and their parents. For those who were incapable of consenting, assent was sought and their substitute decision-maker signed the informed consent.

Eighty-six participants with a clinical diagnosis of ASD, made by child psychiatrists based on the DSM-5, and 39 TDC initially joined the study. The ASD diagnosis in all participants in the ASD group were further confirmed by the Autism Diagnostic Observation Schedule, ADOS [24, 25] and Autism Diagnostic Interview-Revised, ADI-R [26, 27].

Parents of all participants received an interview by the senior author (H.-Y.L.) using the Kiddie-Schedule for Affective Disorders and Schizophrenia-Epidemiological Version (K-SADS-E) for DSM-5 [28], to evaluate co-occurring psychiatric disorders in patients and to ensure that TDC were free of any mental health issues. Exclusion criteria for all participants in the ASD group included: Any acute or unstable medical illness; history of psychosurgery or head trauma; any active grand mal seizures in the past one year; known genetic causes contributing to ASD or ID; a history of bipolar, psychotic or substance use disorders; current suicidal ideation; pregnancy.

In addition to clinical assessments, all participants received the intellectual assessments using the Wechsler Intelligence Scale—4th Edition, either the Wechsler Adult Intelligence Scale-IV (WAIS-IV; for those aged 16 and above) [29] or the Wechsler Intelligence Scale for Children-IV (WISC-IV; for those aged between 6 and 16) [30], and Leiter International Performance Scale-Revised, Leiter-R [31]. Their parents completed several scales to estimate participants’ behaviors and function, including the Social Responsiveness Scales, SRS [26], for autistic traits, the Vineland Adaptive Behavior Scales, VABS [32, 33], for adaptive function (adaptive behavior composite), and the Behavior Rating Inventory of Executive Function, BRIEF [34], for daily life executive function.

After the quality control steps detailed as follows, 65 participants with ASD (6 females, age 16.6 ± 5.9 years) and 38 TDC (8 females, age 17.3 ± 5.6 years) were included in the final dMRI analysis (Table 1). Among those excluded, 14 participants with ASD failed to complete the dMRI scan; 7 participants with ASD and 2 TDC exhibited excessive motion during the dMRI scan.

**Table 1:** Demographic data and clinical features of participants

	Typically developing control (TDC) *n* = 38		Intellectually able ASD (ASD-IA) *n* = 34		Intellectually impaired ASD (ASD-II) *n* = 31		*P* value	Post hoc test
	Mean	SD	Mean	SD	Mean	SD		
Sex (M:F)	30:8		30:4		29:2		0.20^a^	–
Age (years)	17.3	5.65	15.9	5.46	17.3	6.41	0.55^b^	–
RMS (mm)	0.399	0.197	0.407	0.184	0.463	0.216	0.40^c^	–
Total outliers (%)	1.28	0.69	1.48	0.72	1.63	0.95	0.15^c^	–
Medication (Y:N)	0:38		14:20		9:22		0.31^a^	–^d^
Comorbidity (Y:N)	0:38		26:8		24:7		0.93^a^	–^d^
ICV (cm^3^)	1604.7	142.1	1588.8	114.5	1626.2	104.7	0.47^b^	–
*Cognition and symptoms*								
FSIQ (WISC/WAIS-IV)	112.2	11.7	104.5	14.2	65.7	13.0	< 0.001^c^	ASD-II < ASD-IA, TDC
NVFIQ (Leiter-R)	121.6	10.2	115.0	18.3	77.6	26.3	< 0.001^c^	ASD-II < ASD-IA, TDC
VABS-ABC	113.5	15.3	89.2	15.1	66.7	9.6	< 0.001^c^	ASD-II < ASD-IA < TDC
BRIEF-GEC	92.3	19.1	155.3	28.5	152.5	25.5	< 0.001^c^	ASD-II, ASD-IA > TDC
SRS-Total	18.0	10.4	87.6	27.0	93.8	23.3	< 0.001^c^	ASD-II, ASD-IA > TDC
ADOS-2 CSS	N/A	N/A	5.0	2.1	6.8	2.1	0.002^c^	ASD-II > ASD-IA^d^

*RMS* Relative root-mean-square of framewise displacement during diffusion MRI, *ICV* Intracranial volume, *FSIQ* Full-scale intelligence quotient, *WISC-IV* Wechsler Intelligence Scale for Children—4th Edition, *WAIS-IV* Wechsler Adult Intelligence Scale—4th Edition, *NVFIQ* Nonverbal full-scale intelligence quotient, *Leiter-R* Leiter international performance scale-revised, *VABS-ABC* Vineland adaptive behavior scales adaptive behavior composite, *BRIEF-GEC* Behavior rating inventory of executive function global executive composite, *SRS-Total* Social responsiveness scales total raw score, *ADOS-2 CSS* Autism diagnostic observation schedule 2 calibrated severity score

^a^Chi-square; ^b^ANOVA; ^c^Kruskal–Wallis; ^d^comparison between ASD-IA and ASD-II

The whole ASD group (hereinafter ASD-Whole) was categorized into two subgroups: Intellectually Able (hereinafter ASD-IA; those with Wechsler’s full-scale IQ, FSIQ, and adaptive behavior composite > 85, *n* = 34) and Intellectual Impairment (hereinafter ASD-II; those with adaptive function and/or FSIQ < 85, 1.5 standard deviations lower from the TDC norm; *n* = 31). The cutoff for “Intellectual Impairment” was defined because autistic children with borderline IQ (i.e., 70–84) have similar developmental trajectories to those combined with intellectual disabilities [35]. Adaptive function was included in the definition, because intelligence alone is imprecise to predict functional abilities of autistic individuals [36]. Moreover, we intentionally did not adopt the terms “high/low-functioning” herein to preclude prejudicial and inaccurate descriptions [36, 37].

In the ASD-II subgroup, there were 24 individuals with intellectual and cognitive impairment but fair verbal expression capacity (hereinafter ASD-II-Only); 15 out of these 24 ASD-II-Only had FSIQ < 70. The other 7 participants in ASD-II had an MV status (hereinafter ASD-MV; defined by the effective use of < 30 words in verbal expression as reported by their parents [19]). Additional file 1: Table S2 presents the features of the ASD-II-Only and ASD-MV subgroups.

Seven participants with ASD received ADOS Module 1; four received Module 2; twenty-four received Module 3; and thirty received Module 4. We further transformed an ADOS-2 algorithm total raw score [38] to a standardized calibrated severity score (CSS) [39, 40] to allow for the cross-module analysis.

### MRI acquisition and data preprocessing

MRI data were collected on a Siemens MAGNETOM Prisma 3-T MRI with a 64-channel phased-array head/neck coil. High-resolution MPRAGE T1-weighted structural brain images were acquired sagittally using the following parameters: repetition time (TR) = 2000 ms, echo time (TE) = 2.43 ms, inversion time = 920 ms, flip angle = 9°, acquisition matrix = 256 × 256, slice thickness = 0.9 mm, in-plane resolution = 0.9 mm isotropic. Multi-shell DWIs were acquired using the multi-band accelerated echo-planar imaging sequence developed at CMRR [41] with the following parameters: 2.2-mm isotropic voxel, TR/TE = 2238/86 ms, multi-band acceleration factor = 4, number of diffusion gradient directions = 19/30/90/60 at *b* = 0/350/1000/3000 s/mm^2^, respectively. A pair of single *b* = 0 volumes with opposing phase encoding polarities were acquired for the correction of image distortion and motion (as described below). Support procedures for participants’ MRI scans are detailed in Supplement.

DWI data preprocessing included denoising [42], Gibbs ringing removal [43], corrections for image distortions induced by eddy currents and susceptibility effects, inter-volume and slice-to-volume movements [44–46], bias field [47]; these steps were performed using MRtrix3^1^ [10] and FSL^2^ [48]. Quality assessments were performed to exclude the raw DWI data with artifacts or in-scanner motion (based on the average root-mean-square displacements between DWI volumes, relative RMS, > 1 mm). We also assessed the percentage of total outliers (indicating the signal loss) in each participant’s DWI data. The preprocessed DWI data of 38 TDC and 65 autistic participants were upsampled and eventually analyzed using MRtrix3’s FBA described in the next section with recommended pipeline and parameters [10]. We also conducted complementary analysis, using the metrics FA and MD based on the diffusion tensor model, to investigate how specific the FBA results were (detailed in Supplement). The codes of the detailed preprocessing and DWI modeling are available at https://osf.io/vskj7/.

### Metrics and statistics of FBA

The multi-shell multi-tissue constrained spherical deconvolution was applied on each upsampled DWI to compute fiber orientation distributions (FODs) of WM and tissue compartments of GM and cerebrospinal fluid [49], followed by intensity normalization to correct for compartmental inhomogeneities [50]. A study-specific FOD template was created from all TDC and ASD-Whole participants using the FOD-guided registration [51], followed by FOD segmentation [52] to generate the template fixels (FOD threshold = 0.06). All participants’ fixel-wise measures were computed and mapped onto the corresponding template fixels. The fixel-wise metrics include: fiber density (FD), being approximately proportional to the intra-axonal volume fraction of specific fiber bundles in a voxel given typical human dMRI at high *b* values [53, 54]; fiber cross section (FC), measuring the entire macroscopic/volumetric change in the transverse plane section of a local fiber bundle [12]; a combined measure of FD and FC (FDC), quantifying the overall “connectivity” via microscopic density and macroscopic cross-sectional area of a fiber bundle (detailed interpretations in [12]). Since being provided at the fixel level, these FBA metrics are directly relevant to the underlying axonal structure within voxels. By contrast, the voxel-averaged nature of DTI-derived FA is an indirect and non-specific measure of fiber microstructure, and can be erroneous in many voxels containing crossing fibers [9].

We performed the statistical analysis of whole-brain fixel-wise metrics using the general linear model (GLM) incorporated with the connectivity-based fixel enhancement (CFE) approach, which implements tract-specific smoothing and thus improves test statistics of the fixel data [55]. A whole-brain tractogram was generated using the template FODs, post-processed using spherical deconvolution informed filtering of tractograms [52], and then used to compute fixel-to-fixel connectivity required for fixel data smoothing and enhanced statistics [55]. Two types of analyses were conducted as follows:

- Categorical analysis—As described previously, our study cohort includes two main groups (TDC and ASD-Whole) and four ASD subgroups (ASD-IA, ASD-II, ASD-II-Only, and ASD-MV) subdivided from the ASD-Whole. Whole-brain fixel-wise differences were tested between pairs of these groups, including TDC versus ASD-Whole/ASD-IA/ASD-II, respectively, and additionally, ASD-IA versus ASD-II and ASD-II-Only versus ASD-MV. With the limited sample size, the contrast of ASD-MV with ASD-II-Only was intended for initial investigations of the effects of verbal expression capabilities but should be considered preliminary.
- Dimensional brain–behavior analysis—Mass univariate GLMs for the whole sample (ASD-Whole plus TDC) were separately constructed to investigate which fixel (dependent variable) in the brain could be predicted by each of the following independent variables, including nonverbal full-scale IQ (NVFIQ) based on the Leiter-R, adaptive behavior composite, the SRS total raw score, and global executive composite (GEC) on the BRIEF. A behavior-by-diagnosis (TDC vs. ASD) interaction term was also included in these models. Notably, we focused the brain–behavior dimensional analysis on the fluid intelligence (Leiter’s NVFIQ) because individuals in the ASD-MV subgroup cannot complete Wechsler’s verbal intelligence assessment. Another consideration was worth noting: Although the SRS manual provides T-scores for clinical screening use, the raw score is commonly used in research to estimate impairment in social functioning in people with ASD [56]. In addition, within the ASD-Whole group, we also undertook a mass-univariate GLM to investigate the brain–behavior relationship with an independent variable of ADOS-2 CSS.

For both analyses above, the nuisance variables included participant’s sex, age, medication, ICV, and relative RMS. Considering the potential age effects on behavior [57] and brain metrics [58, 59], we also additionally tested whether there were correlations between the age and aforementioned behavior/cognition variables, as well as whether there was an age diagnosis effect on FBA metrics. All these age-related tests yielded null results. The nonparametric permutation-based testing was the statistical method used to correct the family-wise error (FWE) [60, 61] and the corrected FWE *P* value (hereinafter *P-FWE*) was assigned to each fixel after CFE [55] over 5,000 permutations. The outcomes for these two analyses reported in the Results section below were considered statistically significant when per-fixel *P-FWE* < 0.05.

To identify the associated WM anatomy with fixels, we used TractSeg to produce labeled WM fiber bundles [62]. When an identified fixel was located close to the GM or there were no major WM tracts nearby, we applied: (a) the cerebral AAL atlas [63] and the cerebellar SUIT atlas [64] to obtain neuroanatomical labels; (b) Yeo’s 7-network parcellation [65, 66] to designate the functional organization to which these fibers belong.

## Results

### Demographics

As shown in Table 1, all three groups (TDC, ASD-IA, and ASD-II) had comparable distributions of sex, age, relative RMS, percentage of total outliers (signal loss), and intracranial volume (*P* > 0.05). The ratio of medication uses and co-occurring psychiatric disorders was similar between ASD-IA and ASD-II (*P* > 0.05; detailed in Additional file 1: Table S1). With significant differences in Kruskal–Wallis test (*P* < 0.001), post hoc tests showed that the ASD-IA and TDC groups were matched for intellectual function measured by Wechsler’s and Leiter’s batteries. The ASD-IA group had intermediate levels of overall adaptive function in-between the ASD-II and TDC groups. ASD-IA and ASD-II had worse executive function than TDC. ASD-IA had milder autistic symptoms based on both the SRS and ADOS-2 CSS. Additional file 1: Fig. S1 presents the distributions of the intelligence, adaptive function, and symptoms for each subgroup.

### Categorical comparisons

**Fig. 1:**
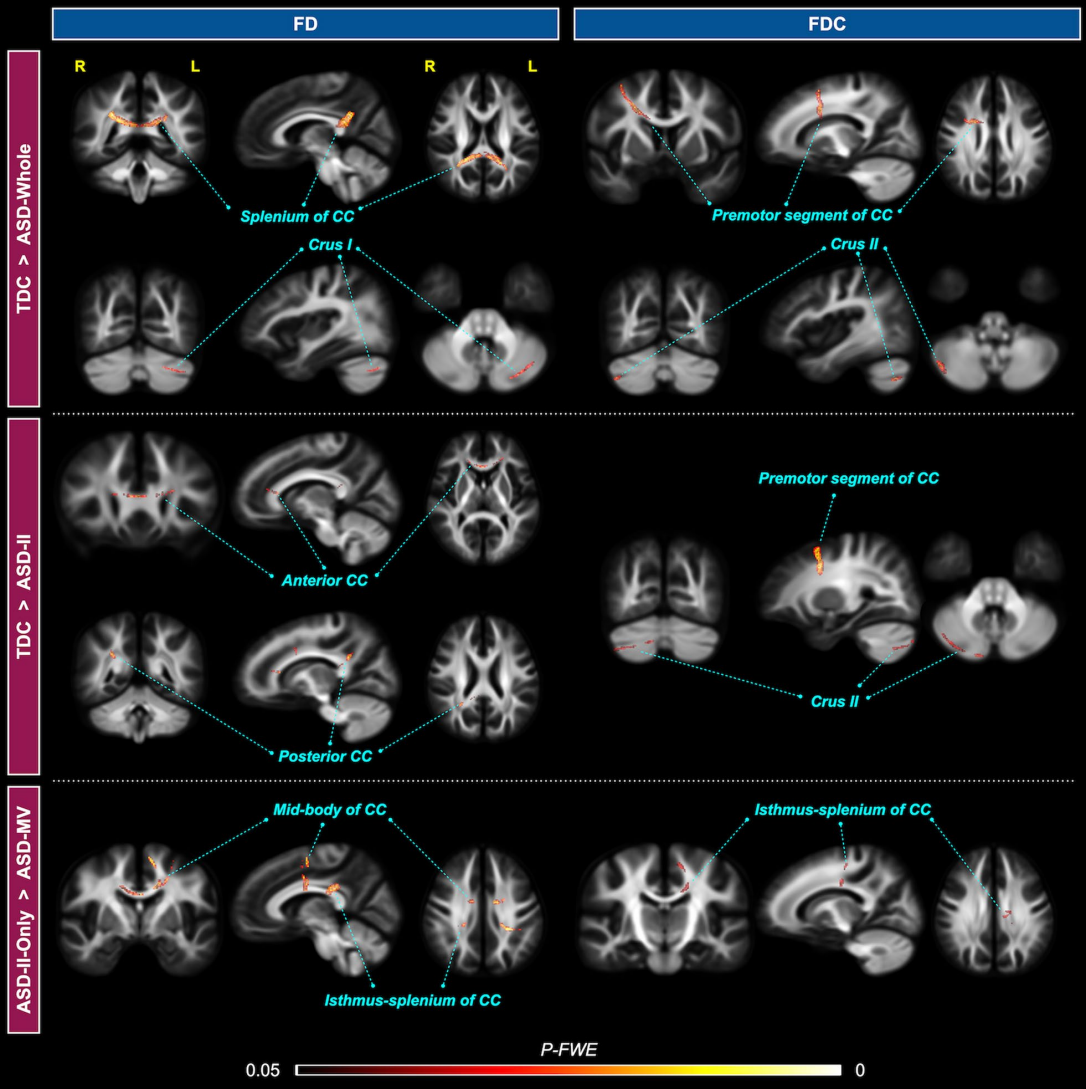
Results of categorical comparisons from the fixel-based analysis. White matter tract segments that have significant group differences in FD (left column) and FDC (right column) metrics are displayed and colored by the family-wise error corrected *P* value (*P-FWE*). Upper block – TDC > ASD; middle block – TDC > ASD-II; bottom block – ASD-II-Only > ASD-MV

The ASD-Whole group, relative to the TDC, had smaller FD in the premotor segment and splenium of CC, alongside the left cerebellum Crus I (default-mode network), and smaller FDC in the right cerebellum Crus II (frontoparietal network; *P-FWE* < 0.05; Fig. 1, top row). Compared to the TDC group, ASD-II had smaller FD in the anterior (interconnecting bilateral prefrontal cortex) and posterior (part of the isthmus-splenium), and showed smaller FDC in the CC segment interconnecting bilateral premotor cortex, as well as in the right Crus II (frontoparietal network; *P-FWE* < 0.05; Fig. 1, middle row). Within the ASD-II subgroup, participants with ASD-II-Only, relative to ASD-MV, had greater FD in the middle segment and isthmus-splenium of CC (*P-FWE* < 0.05). Similar to the FD result, the ASD-II-Only subgroup had greater FDC in the isthmus-splenium of CC than the ASD-MV subgroup (*P-FWE* < 0.05; Fig. 1, bottom row). There was no statistically significant difference in FBA metrics between the TDC and ASD-IA groups, or between the ASD-IA and ASD-II groups (*P-FWE* > 0.05).

Given the spatial overlap, we extracted mean FD values from the common area of CC-nium shared between ASD-Whole vs. TDC and ASD-II vs. TDC. Additional file 1: Fig. S2 illustrates distributions of this FD which showed that ASD-IA had a higher mean and smaller standard deviation of FD (0.728 ± 0.036) than ASD-Whole (0.725 ± 0.043) and ASD-II (0.722 ± 0.050), respectively. Together with its smaller sample size than that of ASD-Whole, this data feature results in null statistically significant differences between ASD-IA and TDC, alongside ASD-II, respectively (see detailed interpretations in Supplement).

### Dimensional brain–behavior relationship

**Fig. 2:**
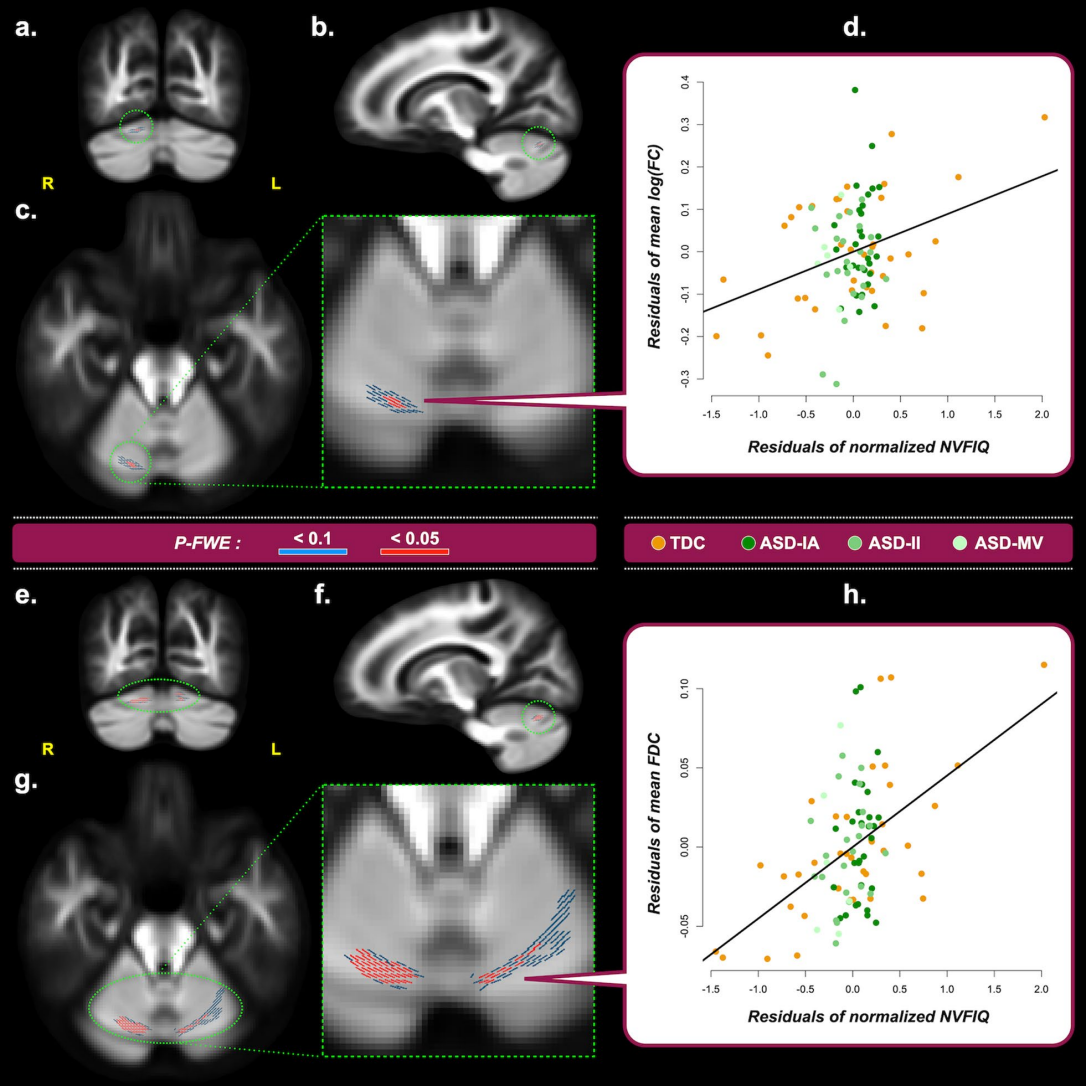
Correlations between fixel metrics and NVFIQ, derived from the dimensional analysis of the entire study cohort. Upper block – Panels **a**–**c** show the fixels where the correlation of log(FC) and Leiter-R’s nonverbal full-scale IQ (NVFIQ) reach statistical significance from the coronal, sagittal, and transverse view, respectively. A zoomed image of **c** is displayed with the green dashed border. Fixels are colored in red for *P-FWE* < 0.05; fixels colored in blue indicate *P-FWE* < 0.1 and are used to assist identification of the associated brain structure. **d** The scatter plot shows the residuals of the mean log(FC) on the vertical axis and of the normalized NVFIQ on the horizontal axis. Only all fixels that reach *P-FWE* < 0.05 are considered in the plot. Lower block – The format in Panels **e**–**h** is the same as the upper block, except that the results are obtained from the analysis of FDC and NVFIQ. Acronyms – *R* Right, *L* Left, *P-FWE* Family-wise error corrected *P* value

**Fig. 3:**
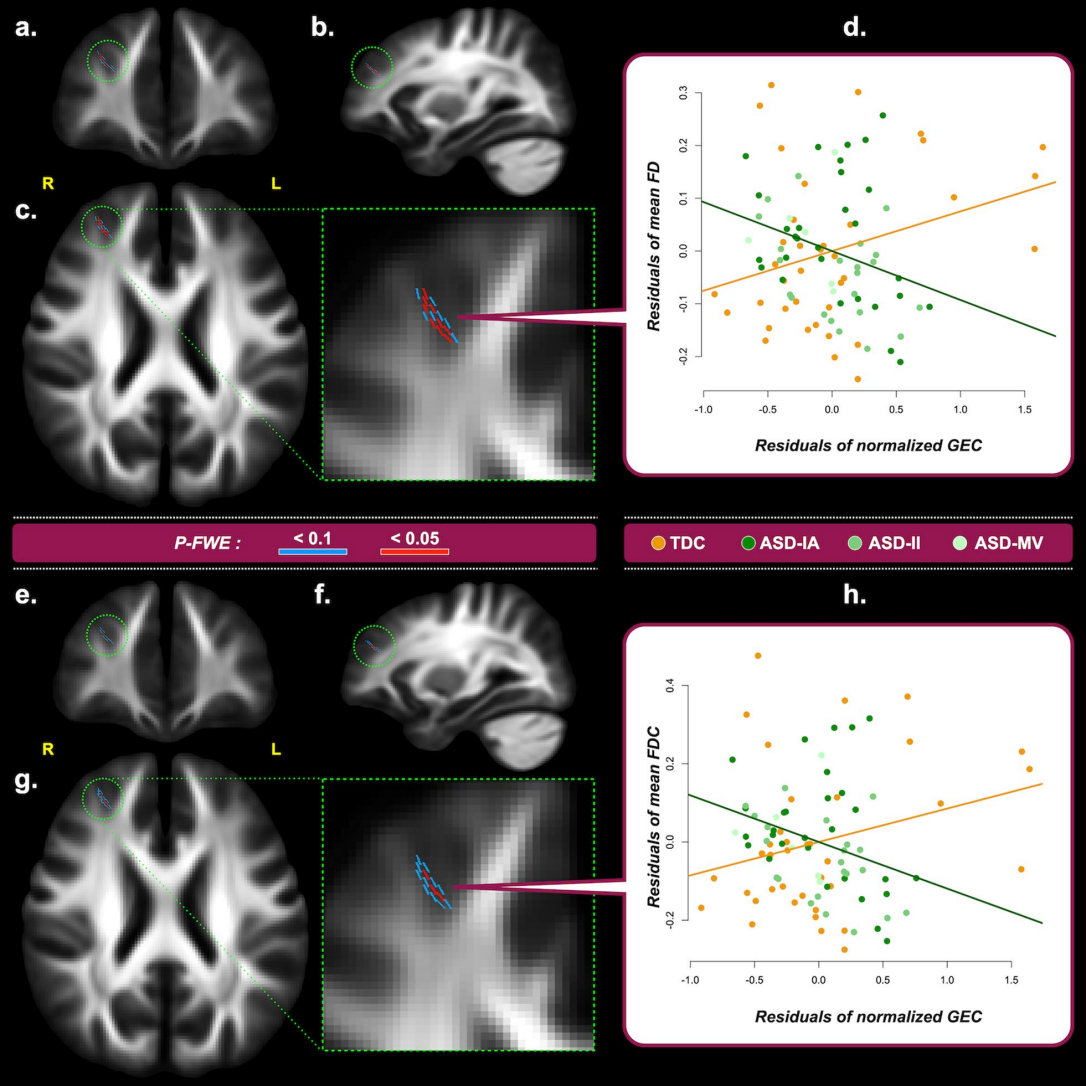
Correlations between fixel metrics and Global Executive Composite (GEC), derived from the dimensional analysis of the entire study cohort. Upper block – Panels **a**–**c** show the fixels where the correlation of FD and GEC × diagnosis interactions reach statistical significance from the coronal, sagittal, and transverse view, respectively. A zoomed image of **c** is displayed with the green dashed border. Fixels are colored in red for *P-FWE* < 0.05; fixels colored in blue indicate *P-FWE* < 0.1 and are used to assist identification of the associated brain structure. **d** The scatter plot shows the residuals of the mean FD on the vertical axis and of the normalized GEC on the horizontal axis. The lines are the regression lines fitting the data, with the orange being for TDC and the green being for ASD. Only all fixels that reach *P-FWE* < 0.05 are considered in the plot. Lower block – The format in Panels **e**–**h** is the same as the upper block, except that the results are obtained from the analysis of FDC and GEC. Acronyms – *R* Right, *L* Left, *P-FWE* Family-wise error corrected *P* value

From the whole-sample GLMs, Fig. 2 shows that NVFIQ significantly positively correlated with FC (Fig. 2a–d) and FDC (Fig. 2e–h) of fiber bundles in the cerebellum lobule VI (ventral attention/salience network (*P-FWE* < 0.05). Significant GEC $\times$ diagnosis interactions were identified at the fibers at the GM-WM borders of the right dorsolateral prefrontal cortex (DLPFC, middle frontal gyrus subregion belonging to ventral attention/salience network; *P-FWE* < 0.05; Fig. 3a, b for FD, 3e–g for FDC). Specifically, GEC positively correlated with the DLPFC FD/FDC in TDC, while this brain–behavior association was negative in the ASD-Whole group (Fig. 3d, h). We did not find significant correlations or diagnosis $\times$ behavior interactions of the SRS and VABS with fixel metrics (*P-FWE* > 0.05).

**Fig. 4:**
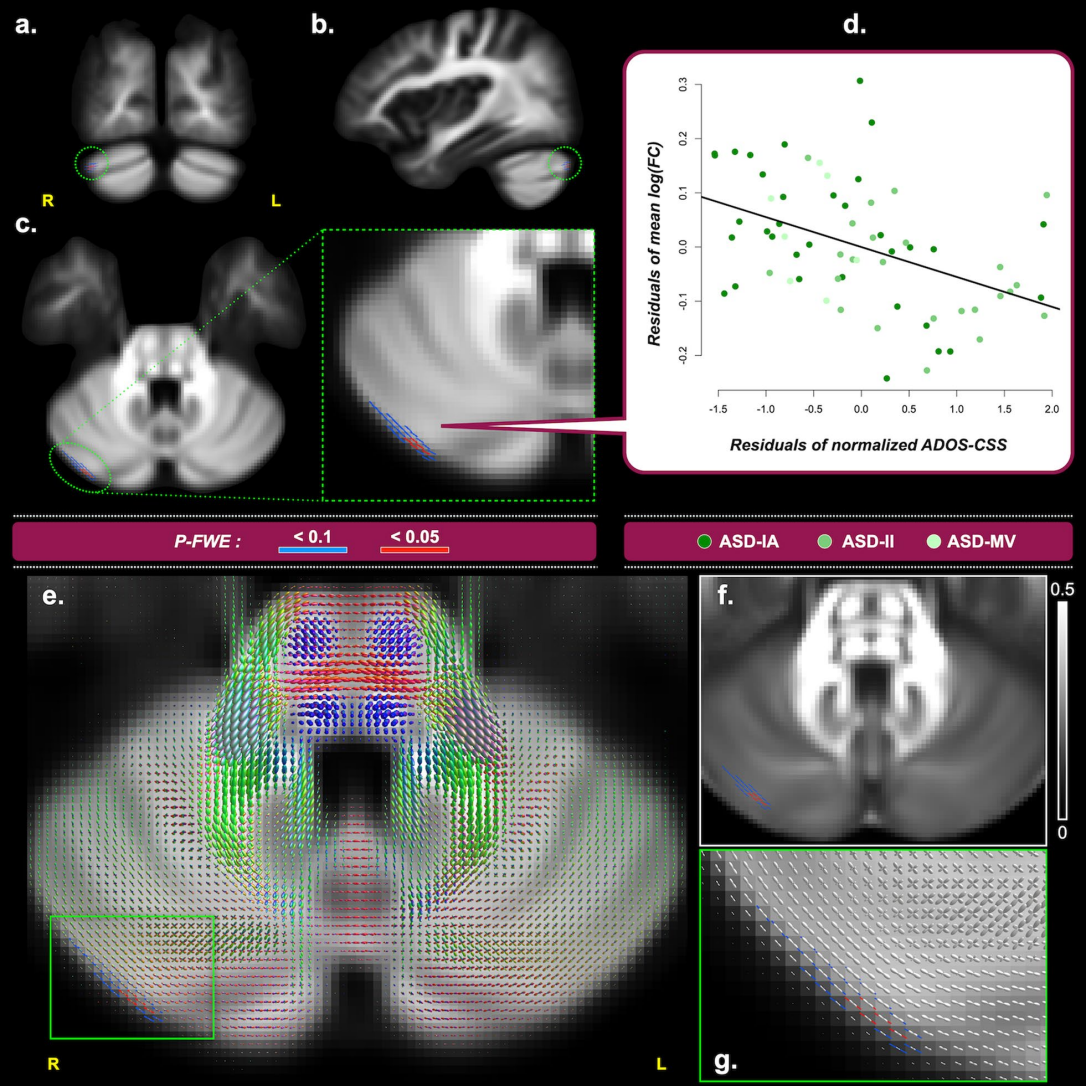
Correlations between fixel metrics and Autism Diagnostic Observation Schedule 2 Calibrated Severity Score (ADOS-CSS), derived from the dimensional analysis of the entire ASD cohort (i.e., ASD-Whole). Upper block – Panels **a**–**c** show the fixels where the correlation of log(FC) and ADOS-CSS reach statistical significance from the coronal, sagittal, and transverse view, respectively. A zoomed image of **c** is displayed with the green dashed border. Fixels are colored in red for *P-FWE* < 0.05; fixels colored in blue indicate *P-FWE* < 0.1 and are used to assist identification of the associated brain structure. **d** The scatter plot shows the mean log(FC) residuals on the vertical axis and the normalized ADOS-CSS residuals on the horizontal axis. Only all fixels that reach *P-FWE* < 0.05 are considered in the plot. Lower block – **e** shows an axial slice of the group average FOD image. **f** shows the FA map of the same slice of (**e**). **g** is a zoomed region of (**e**), showing the microstructural organization of axonal fibers around the significant fixels within the cerebellum, which could not be reliably studied using tensor-based voxel-based analysis. Acronyms – *R* Right, *L* Left, *P-FWE* Family-wise error corrected *P* value

The within-ASD GLM yielded that autistic individuals’ ADOS-2 CSS negatively correlated with WM FC in the right cerebellum Crus I (default-mode network; *P-FWE* < 0.05; Fig. 4a–d). Figure 4f shows that cerebellar FA values are low (~ 0.2), which cannot serve meaningful analysis. Figure 4e, g illustrates that the cerebellar FBA FODs constituted a distinct structural organization that statistical analysis can be based upon.

### Robustness testing

This study adopted three strategies to test the robustness and specificity of current findings. First, during the generation of the template fixels, thresholding the amplitude of the FOD lobe is required to minimize the misalignment of fixels across subjects; such an effect is particularly pronounced at the GM-WM interface where registration could be imperfect [55]. Hence, to ensure that the results were not driven by the choice of the FOD threshold, three additional FOD cutoff values of 0.04, 0.08, and 0.10 were used to construct the fixel template, and all the subsequent analyses including categorical comparisons and dimensional analysis were repeated. The main results in the categorical analysis above remained largely the same across different FOD thresholds (Additional file 1: Fig. S3). The repeated dimensional analysis also showed consistent findings in the correlated brain structures, despite some variations in fixel locations (Additional file 1: Fig. S4). Second, a more stringent significance level of *P-FWE* < 0.01 was applied to test the current results. Few of the results met this strict criterion and they had reduced spatial extent of fixels (Additional file 1: Figs. S5, S6). Lastly, VBA was performed with all the analyses repeated based on FA and MD. VBA neither replicated any of the results obtained from FBA nor provided additional findings (Additional file 1: Fig. S7).

## Discussion

Using the current state-of-the-art dMRI sequence and FBA in a cohort with ASD across the functional spectrum, we found that ASD-associated alterations in fixel-based metrics, as identified in ASD-Whole vs. TDC, were largely driven by those combined with intellectual impairment and/or potentially by minimally verbal status (Additional file 1: Fig. S2). In addition to the altered CC, consistent with the earlier literature [7, 13], we provide initial evidence suggesting that cerebellar WM also categorically and dimensionally links with the clinical presentation of ASD. Our findings highlight an essential to include autistic participants with wide-range functional levels in research, to enable a greater neurobiological understanding of the variety of functioning and cognitive profiles that manifest across the autism spectrum.

The present categorical comparison between ASD-Whole and TDC identified reduced FD in the splenium section of the CC and reduced FDC in the premotor segment of the CC, consistent with an earlier FBA report on intellectually able individuals with ASD [13]. These alterations of the CC fibers are compatible with previous dMRI and other modalities literature suggesting impaired interhemispheric connection might contribute to idiosyncratic socio-emotional behaviors and sensorimotor integration associated with ASD [7, 67, 68]. Intriguingly, the finding of ASD-associated reduced connectivity of premotor CC may echo an earlier study showing that microstructural property of the premotor corticospinal tract is linked with repetitive and restricted behaviors [69]. However, the altered CC microstructure appears not specific to ASD [70]. This long-standing challenge in finding disorder-specific neuroimaging biomarkers might be better disambiguated with an advanced approach of linking microscale genomic and macroscale neuroimaging observations [71].

In addition, we identified that the ASD-Whole group showed altered geometries of fiber-bundle populations in the cerebellum Crus I/II. Moreover, autistic symptoms based on the ADOS correlated with the fiber bundle morphometry in the right Crus I. The left-side Crus I/II supports the socio-emotional inferences and motion-related imitation [72, 73], whereas the right counterpart subserves the complex socio-emotional reasoning and sensorimotor integration [74, 75] in neurotypical people. The GM morphometry and function of Crus I/II are reported to be involved in impaired socio-emotional [75] and language processes, alongside repetitive behaviors [74] in people [76] and mice models [77, 78] with ASD. Intellectually able youth with ASD also have specific developmental changes in GM morphometry at these loci [59, 75]. Moreover, the identified Crus I and Crus II regions are separately functionally located in the default-mode and frontoparietal networks, of which altered cerebral activity and connectivity have been observed in ASD [79, 80]. Taken together, consistent with the functional MRI literature, we provide the first empirical evidence to imply that Crus I/II WM essentially links with autistic conditions. However, whether cerebellar lateralization plays a role in ASD-associated fiber pathology awaits further confirmation.

When comparing ASD-IA with TDC, with the similar sample size and demographic features to the participants of Dimond et al*.*’s work [13], we did not replicate the significant ASD-associated WM differences. Conversely, the ASD-II vs. TDC differences were similar to ASD-Whole vs. TDC differences in the CC-splenium (based on FD) and right Crus II (based on FDC). Despite the preliminary nature due to a small sample size of ASD-MV, the difference between ASD-II-Only and ASD-MV also was located at the posterior CC (isthmus-splenium). This echoes findings from a structural MRI study on neurotypical adults [81], a dMRI study [82] on adults with dyslexia, and a dMRI report on the neurotypical development of language in toddlers [83], suggesting an essential role of splenial neuroanatomy in verbal and language processing. As shown in Additional file 1: Fig. S2, our findings suggest that ASD-associated alterations in the geometry of fiber-bundle populations, as identified by ASD-Whole vs. TDC, are mainly driven by the fiber pathology in those autistic people with lower intellectual/adaptive function. This uncovering might help reconcile parts of inconsistency in the previous FBA [13, 14] and other MRI literature [84]. This is because these neuroimaging studies chiefly based on intellectually able individuals [20] tend to show smaller effect sizes [85]. Our results also highlight the imperativeness of having research practices be inclusive of all autistic individuals on the spectrum, especially the understudied population with developmental disabilities [21, 22], in order to address a gap of better characterizing these diverse autistic samples and development of more individualistic targeted plan accordingly [19, 20, 23].

The dimensional brain–behavior analysis showed that the positive correlation between fluid intelligence and FC/FDC metrics in the cerebellum lobe VI existed cutting across ASD and TDC as a continuum. The higher the nonverbal intelligence is, the stronger the fiber-bundle populations are. Earlier functional MRI studies reported that the cerebellar VI is involved in motor processing, and nonmotor cognitive processing is implicated in language and working memory in ASD [86–89]. This posterolateral cerebellar area also supports the foundational function of hierarchical cognitive control such as mental arithmetic [90]. This lobule also participates in the ventral attention/salience network [91], whose cerebral connectivity links with neurotypical individuals’ fluid reasoning capacity [92]. Taken together, the current result suggests the essential role of the posterior cerebellum in contributing to human fluid cognition, regardless of the neurodiverse status.

Moreover, we identified a diagnosis-specific relationship between the summarized measure of daily life executive function and the FC/FDC in the GM-WM borders of the right DLPFC in the ventral attention/salience network. Individuals with ASD usually have a broad executive dysfunction that is relatively stable across development [93]. The DLPFC is one of the primary areas that support executive function processing such as planning and working memory [94, 95]. This prefrontal area plays an essential role in executive dysfunction associated with ASD across ages [96]. Moreover, the ventral attention/salience network appears to link with ASD [97] and self-control [98] in different directions. ASD has poor delineation of the GM-WM boundary at the DLPFC [99, 100]. Collectively, while future studies are required to establish the relationship between the fiber-bundle populations and the differentiation of the GM-WM boundary, it is likely that altered neurodevelopmental processes involving WM fibers at the GM-WM boundary contribute to executive dysfunction commonly observed in ASD.

The capability of FBA to quantify fiber-specific measures of micro-/macrostructural alterations has been shown to be more beneficial for cerebral WM over VBA [11, 101]. Advancing previous findings based on the DTI model [21, 22] and advanced model of structural T1 image [99], our study reveals that the cerebellum and the WM underneath as well as the GM-WM junction at the DLPFC are involved in ASD pathology and associated cognition. The supplementary analysis (Additional file 1: Figs. S3, S4, S5 and S6) not only supports the robustness of these findings, but also suggests that provided the equivalence with the present dMRI data quality in combination with multi-tissue modeling [49], FBA could be advantageous to detect small-yet functionally meaningful alterations in voxels containing complex arrangement of axonal compartments, such as at the GM-WM partial volume voxels or cerebral/cerebellar parenchyma. Notably, these findings were only observed using FBA, whereas VBA based on diffusion tensor measures did not yield any significant results. This discrepancy may originate from that tensor-based metrics are prone to the effects of crossing fibers and therefore more susceptible to noise contamination at those brain regions [102]. Among other explanations of sampling heterogeneity [17], sex-mediated differences [14, 103], and head motion [16], the current strength of using advanced analysis technique might also partly account for the null finding in the uncinate fasciculus, inconsistent with earlier meta- [5] and mega-analyses [70] mainly based on tensor-based metrics. This WM tract is especially liable to false-positive findings because of the crossing fibers [104, 105].

## Limitations

First, the sample had a relatively wide age range. Although our additional age-related tests yielded null results, we cannot exclude residual developmental effects on the current findings. Second, the present sample was male predominant, limiting any formal tests on sex-related differences [106]. Third, we recruited participants from outpatient clinics, with many showing psychiatric comorbidities commonly associated with ASD (such as ADHD and anxiety disorders) or/and psychotropic medication uses. On the flip side, this design may render the current results more generalizable to “real-world autism,” as these psychiatric comorbidities may affect up to 70% of individuals with ASD [107]. The intellectually able participants with ASD without (or with restricted proportions of) psychiatric comorbidities in most of earlier neuroimaging literature actually belong to an outlier subgroup on the spectrum [17]. Moreover, there was no difference in ratios of comorbidity and medication utilization between the ASD subgroups, which reduces the doubt that the current results might be driven by such comorbidities. Fourth, our sample size was relatively small, limiting statistical power to detect small between-group effects and paradoxically increasing the risk of inflating effect sizes [108]. In particular, the outcomes related to ASD-MV should be considered preliminary. Lastly, we did not include intellectual impairment-only without ASD as an additional control condition. Scientific progress will benefit from intellectually informed recruitment strategies to discover where ASD and intellectual impairment intersect and part ways.

## Conclusion

In sum, we confirmed that changes in anatomical connections linking hemispheres, as well as cerebellum Crus I/II, might contribute to autism phenotypes. These ASD-associated alterations appear to be mainly driven by autistic individuals with intellectual impairment and potentially further by minimally verbal status. Across the functional spectrum, autistic severity, nonverbal intelligence, and executive function associated with WM fiber-bundle properties in regions where WM pathology based on dMRI are seldom reported. These results highlight that by embracing the inclusion of understudied subpopulations on the spectrum, together with the development of novel neuroimaging methods, we may better reconcile heterogeneity across studies and advance the understanding of the neuropathology of ASD.

## Supplementary Information

**Additional file1:** **Tables S1 and S2. **Supplementary clinical information on participants with ASD. **Fig. S1. **Distributions of clinical assessment scores of the study cohort. **Fig. S2.** Distributions of per participant’s mean fiber density metric at the splenium of the corpus callosum in each group. **Fig. S3 and S4.** Results of FBA under different FOD cutoff values. **Fig. S5 and S6.** Results of FBA under a statistical threshold of P-FWE < 0.01. **Fig. S7.** Results of conventional voxel-based analysis using the diffusion tensor model.

## Data Availability

The data that support the findings of this study are available from the corresponding author, H-YL, upon reasonable request.

## Abbreviations

ADI-R: Autism Diagnostic Interview-Revised
ADOS: Autism diagnostic observation schedule
ADOS-CSS: Autism diagnostic observation schedule 2 calibrated severity score
ASD: Autism spectrum disorder
BRIEF: Behavior rating inventory of executive function
CC: Corpus callosum
DLPFC: Dorsolateral prefrontal cortex
dMRI: Diffusion magnetic resonance imaging
DWI: Diffusion-weighted image
FA: Fractional anisotropy
FBA: Fixel-based analysis
FD: Fiber density
FC: Fiber cross section
FDC: Fiber density and cross section
FOD: Fiber orientation distribution
FSIQ: Full-scale intelligence quotient
GEC: Global executive composite
GLM: General linear model
GM: Gray matter
IA: Intellectually able
ICV: Intracranial volume
ID: Intellectual disabilities
II: Intellectual impairment
K-SADS-E: Kiddie-schedule for affective disorders and schizophrenia-epidemiological version
Leiter-R: Leiter international performance scale-revised
MD: Mean diffusivity
MV: Minimally verbal
NVFIQ: Nonverbal full-scale intelligence quotient
P-FWE: Family-wise error corrected *p* value
RMS: Root-mean-square
SRS: Social responsiveness scales
TDC: Typically developing controls
VBA: Voxel-based analysis
VABS: Vineland adaptive behavior scales
WAIS-IV: Wechsler Adult Intelligence Scale—4th Edition
WISC-IV: Wechsler Intelligence Scale for Children—4th Edition
WM: White matter

## Footnotes

1 https://www.mrtrix.org/.

2 https://fsl.fmrib.ox.ac.uk/fsl/fslwiki/FSL.
