# Supplementary material for: White matter microstructural and morphometric alterations in autism: implications for intellectual capabilities
Source: Mol Autism. 2022 May 18;13:21. doi: 10.1186/s13229-022-00499-1 (PMC9118608; doi:10.1186/s13229-022-00499-1)
Supplement: Supplementary file 1 — Additional file1: Tables S1 and S2. Supplementary clinical information on participants with ASD. Fig. S1. Distributions of clinical assessment scores of the study cohort. Fig. S2. Distributions of per participant’s mean fiber density metric at the splenium of the corpus callosum in each group. Fig. S3 and S4. Results of FBA under different FOD cutoff values. Fig. S5 and S6. Results of FBA under a statistical threshold of P-FWE < 0.01. Fig. S7. Results of conventional voxel-based analysis using the diffusion tensor model. [file 13229_2022_499_MOESM1_ESM.docx]

**Supplementary Material**

**White matter microstructural and morphometric alterations in autism: Implications for intellectual capabilities**

Chun-Hung Yeh, Rung-Yu Tseng, Hsing-Chang Ni, Luca Cocchi, Jung-Chi Chang, Mei-Yun Hsu, En-Nien Tu, Yu-Yu Wu, Tai-Li Chou, Susan Shur-Fen Gau, Hsiang-Yuan Lin

**A. Supplementary Tables**

**Table S1. Comorbidity and Medication Use for ASD-IA & ASD-II**

|  | ASD-IA (n= 34) | ASD-II (n= 31) |
| --- | --- | --- |
| **Comorbidity** | | |
| No comorbidity | 8 | 7 |
| Comorbid ADHD | 20 | 18 |
| Comorbid Anxiety disorder | 8 (5 social anxiety disorder; 1 history of mild agoraphobia; 1 height phobia) | 9 (4 specific phobia; 4 social anxiety disorder; 1 selective mutism) |
| Comorbid tic disorder | 3 | 5 (2 Tourette syndrome) |
| Comorbid OCD | 1 | 1 |
| Comorbid learning disorder | 1 (writing/reading disorder) | N/A |
| Comorbid depressive disorder | 3 (1 history of depression; 1 dysthymic disorder & mild MDD) | N/A |
| Other comorbidities | 1 gender dysphoria  1 ODD | 2 epilepsy (well controlled)  1 ODD |
|  |  |  |
| **Medications** | | |
| Methylphenidate | 13 | 6 |
| Antidepressant | 2 (1 sertraline; 1 fluoxetine) | 1 (sertraline) |
| Valproic acid (mood stabilizer) | N/A | 2 |
| Antipsychotic | N/A | N/A |

Acronym – ADHD: Attention Deficit/Hyperactivity Disorder; MDD: Major Depressive Disorder; OCD: Obsessive Compulsive Disorder; ODD: Oppositional Defiant Disorder.

**Table S2. Demographic data and clinical features of ASD sub-groups**

|  | Intellectual Able ASD (IA, n=34) | |  | Intellectual Impairment with Fair Verbal ASD (II-Only, n=24) | |  | | Intellectual Impairment with Minimally Verbal ASD (MV, n=7) | | | |  | | P-value | Post-hoc test | |
| --- | --- | --- | --- | --- | --- | --- | --- | --- | --- | --- | --- | --- | --- | --- | --- | --- |
|  | Mean | SD |  | Mean | SD |  | | Mean | | SD | |  | |  |  | |
| Sex (M:F) | 30:4 | |  | 22:2 | |  | | 7:0 | | | |  | | 0.61^a^ | - | |
| Age (years) | 15.9 | 5.46 |  | 17.0 | 6.43 |  | | 18.4 | | 6.73 | |  | | 0.60^b^ | - | |
| RMS (mm) | 0.41 | 0.18 |  | 0.50 | 0.23 |  | | 0.33 | | 0.10 | |  | | 0.19^b^ | - | |
| Total Outliers (%) | 1.48 | 0.72 |  | 1.73 | 1.05 |  | | 1.29 | | 0.41 | |  | | 0.59^b^ | - | |
| Medication (Y:N) | 14:20 | |  | 7:17 | |  | | 2:5 | | | |  | | 0.59^a^ | - | |
| Comorbidity (Y:N) | 26:8 | |  | 19:5 | |  | | 5:2 | | | |  | | 0.91^a^ | - | |
| ICV (cm^3^) | 1588.8 | 114.5 |  | 1625.5 | 113.9 |  | | 1628.8 | | 71.3 | |  | | 0.61^b^ | - | |
| *Cognition and Symptoms* | | | | | | | | | | | | | | | | |
| FSIQ (WISC/WAIS-IV) | 104.5 | 14.2 |  | 68.5 | 11.6 |  | 53.4 | | 12.7 | |  | | <0.001^b^ | | IA > II-Only, MV |  |
| NVFIQ (Leiter-R) | 115.0 | 18.3 |  | 80.6 | 25.9 |  | 66.3 | | 27.1 | |  | | <0.001^b^ | | IA > II-Only, MV |  |
| VABS-ABC | 89.2 | 15.1 |  | 69.2 | 8.92 |  | 57.3 | | 5.99 | |  | | <0.001^b^ | | IA > II-Only, MV |  |
| BRIEF-GEC | 155.3 | 28.5 |  | 150.7 | 26.0 |  | 159.3 | | 24.2 | |  | | 0.67^b^ | | - |  |
| SRS-Total | 87.6 | 27.0 |  | 93.5 | 25.0 |  | 95.2 | | 17.0 | |  | | 0.65^b^ | | - |  |
| ADOS-2 CSS | 5.00 | 2.10 |  | 7.42 | 1.89 |  | 4.57 | | 0.79 | |  | | <0.001^b^ | | II-Only > IA, MV |  |

^a^: Chi-square; ^b^: Kruskal-Wallis

Acronyms: RMS: relative Root-Mean-Square of framewise displacement during diffusion MRI; ICV: Intra-Cranial Volume; FSIQ: Full-Scale Intelligence Quotient; WISC-IV: Wechsler Intelligence Scale for Children-4th edition; WAIS-IV: Wechsler Adult Intelligence Scale-4th edition; NVFIQ: Non-Verbal Full-scale Intelligence Quotient; Leiter-R: Leiter International Performance Scale-Revised; VABS-ABC: Vineland Adaptive Behavior Scales Adaptive Behavior Composite; BRIEF-GEC: Behavior Rating Inventory of Executive Function Global Executive Composite; SRS-Total: Social Responsiveness Scales Total Raw Score; ADOS-2 CSS: Autism Diagnostic Observation Schedule 2 Calibrated Severity Score.

**Figure S1.** Raincloud plots showing the distribution of intellectual function, symptoms, executive function, and adaptive function for each subgroup with the 1.5 SD cutoffs below the TDC norm.

**
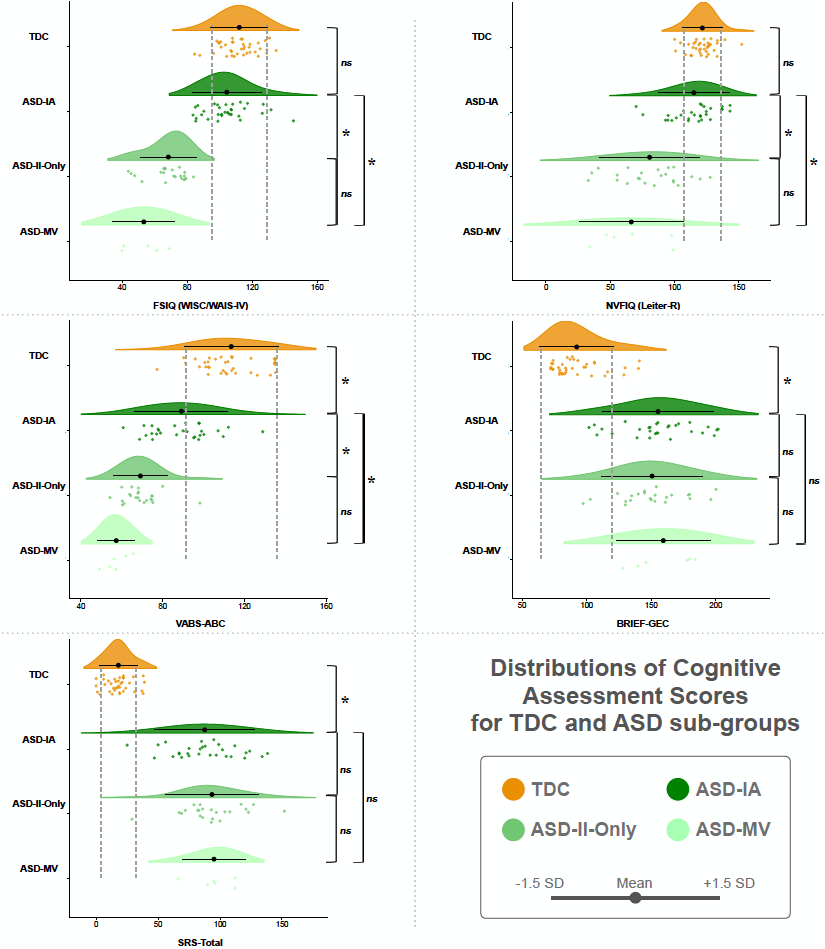
**

**B. Support procedures for MRI scans**

Except the screening survey in clinics, our support strategies for MRI scans were devised and adapted from the published protocol [1]. Specifically, the clinicians in outpatient clinics made a preliminary evaluation to survey whether candidate participants with ASD (especially those with II) might be able to comply with MRI procedures. Following the first-step screening and behavioral assessments, we then shared video and recordings of scanner sounds with each participant (who shows the potential for completing the scans) and their parents two weeks before the scans, in order to help them simulate the scanning environment at home. We also offered an optional one-hour session in a mock scanner within one week before the scan day, if participants or their parents requested or desired the practice.

On the scan day, we would schedule participants 30 minutes to one hour before the formal procedure to help them reduce anxiety by slow exposure to the scan environment, including the room, bed, head coils, and scanner. All participants wore their own MRI-safe clothes without changing into scrubs. After MRI safety checks, their parents, therapist, or our research assistant were allowed to accompany and physically contact participants (touching/patting abdomen or legs or holding hands) during the scanning. This physical company was requested for every participant in the ASD-II subgroup and was optional for the others. Participants’ comfort items were also allowed in the scan room if passing safety checks. Blankets, passive noise-canceling earplugs, soft pads were prepared and opted for individual’s special sensory needs. A movie paradigm, Inscapes, which has shown efficacy to improve compliance in MRI [2], was repeatedly played during T1 and DWI acquisition, while participants were also allowed to freely close their eyes if they disliked the videoclips. We would communicate verbally with the parent of participant with ASD-II or with the participant (with ASD-IA or controls) via the microphone from the control room to ensure their physical and psychological status was good enough for the scans.

**C. Mean fiber density of the splenium of corpus callosum across groups**

The outcomes of the categorical analysis shown in **Figure 1** demonstrated that the significant differences of FBA metrics were found at: TDC > ASD-Whole, TDC > ASD-II, and ASD-II-Only > ASD-MV. When knowing TDC > ASD-Whole, one would expect that TDC should also have greater fixel metrics (e.g. FD) than any ASD subgroups. Thus, the results of no significant differences between TDC and ASD-IA might seem to be contradictory to such expectations. The purpose of this section is to attempt to explain the rationale behind the relevant findings.

**Figure S2** shows the complementary outcomes to **Figure 1**, from which a mask containing “significant fixels” at the splenium of the corpus callosum was derived first. For each (sub)group, the mean and standard deviation of the FD metric within the fixel mask was then computed for each participant, yielding the group distribution of FD. Consistent with **Figure 1**, TDC showed significantly higher mean FD than ASD-Whole. In addition, divided from ASD-Whole, the results suggest that the distribution of ASD-IA “shifted” toward TDC, thus yielding non-significant differences between TDC and ASD-IA.

Likewise, **Figure S2** also explains why there were no differences between ASD-IA and ASD-II: When separated from ASD-Whole, the distribution of ASD-II indeed “shifted” oppositely with ASD-IA. However, although both subgroups had significant differences in their clinical features (Table 1), the alteration in mean FD did not reach the statistical significance. Nevertheless, such a shift toward reduced mean FD resulted in a significantly lower value of ASD-II as compared to TDC, which was consistent with the outcomes of FBA shown in **Figure 1**.

Combining **Figures 1 and S2** therefore leads us to the conclusion that fiber-specific alterations are driven by those combined with II and/or MV, and that it is imperative to include these populations in ASD research to avoid the selection bias.


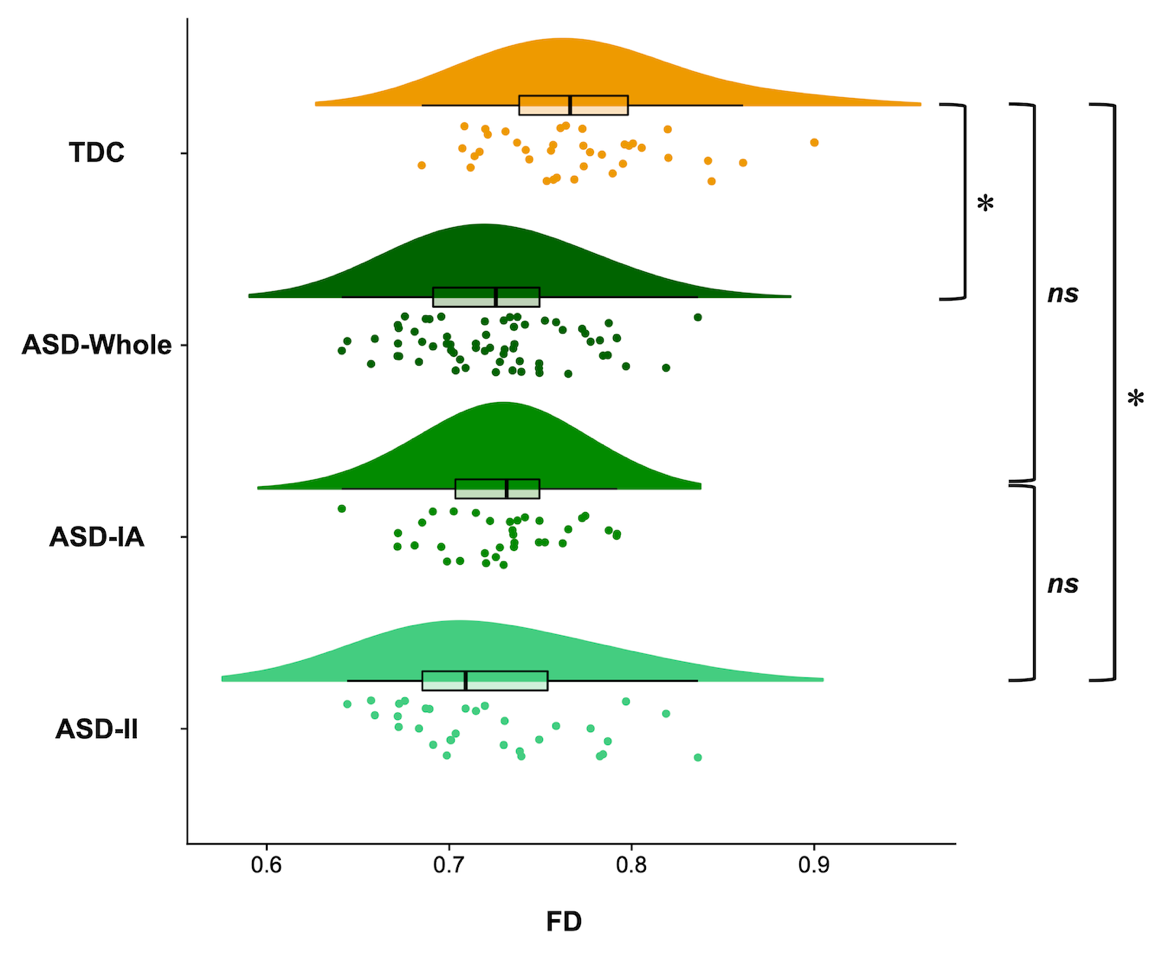


**Figure S2:** Raincloud plots showing the distribution of per participant’s mean fiber density (FD) metric in each (sub)group. FD values were extracted from fixels that showed significant reductions in FD at the splenium of the corpus callosum, which was defined by the spatial overlap between the outcome of TDC vs. ASD-Whole and TDC vs. ASD-II. “*” indicates significant differences of the distribution; “*ns*” denotes that the differences between groups are non-significant.

**D. FBA at different thresholds of FOD peak amplitudes**

The threshold of the FOD peak value defines the minimum amplitude of an FOD lobe to be regarded as a fixel. Hence, lowering the threshold will include small fixels potentially representing a small fraction of fibers presenting in a voxel, at the price of increasing false positive fixels that could come from the effect of noise contamination. Likewise, increasing the threshold will make the FBA focus only on large fixels or major WM fiber bundles, it would however have a detrimental effect as the contribution of fixels below the threshold to the DWI signal will be completely discarded. The default FOD cutoff in *MRtrix3* was set to 0.06 empirically, we therefore provide the results of complementary FBA in this section, in which we adjust the value to 0.04 / 0.08 / 0.10 to ensure that the findings in the current study were not determined by the choice of the threshold. Overall, the FBA outcomes remain largely consistent following the adjustment of the FOD cutoff value. Despite some expected variations in the spatial extent of the significant fixels, no additional anatomical regions were identified.

**Figure S3** shows the results of categorical comparisons under the FOD cutoff values of 0.04 / 0.08 / 0.10. Comparing with the default threshold of 0.06 shown in **Figure 1**, only one brain region where the group differences became non-significant: In TDC versus ASD-II, no significant difference in FD at the posterior CC was detected under the cutoff value of 0.10.


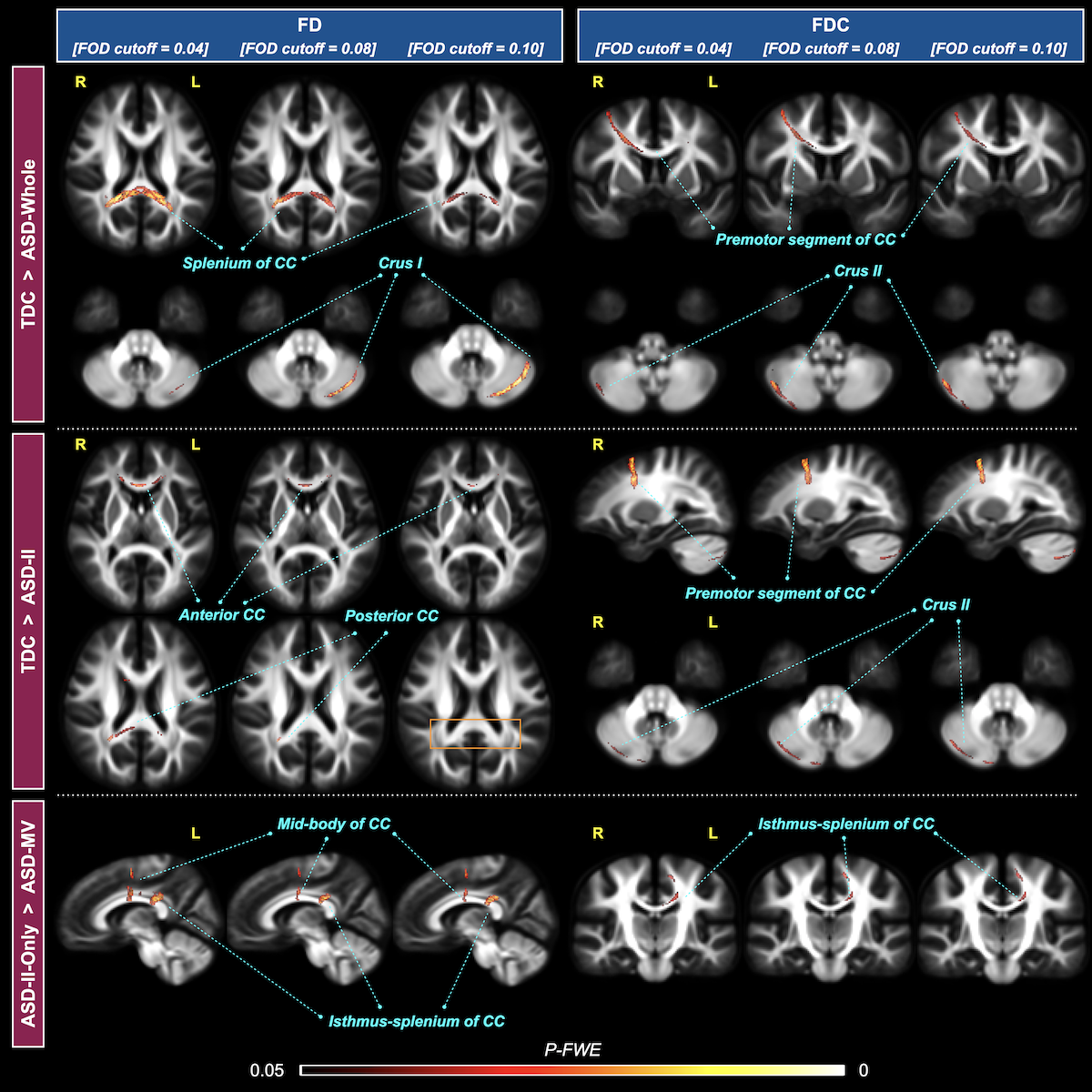


**Figure S3:** Results of categorical comparisons using an FOD cutoff threshold of 0.04, 0.08, and 0.10. Different thresholds were listed from left to right, for the FD (left column) and FDC (right column) metric. Significant fixels are colored by *P-FWE* and overlaid on the FOD template. Upper block ⎯ TDC > ASD-Whole; middle block ⎯ TDC > ASD-II; bottom block ⎯ ASD-II-Only > ASD-MV. The rectangle colored in orange indicates the missing significance comparing to the original results under the FOD cutoff of 0.06 in **Figure 1** of the main content.

**Figure S4** shows that the dimensional brain-behavior analysis under different FOD cutoff values has a similar pattern with the results shown in **Figures 2 and 3**.


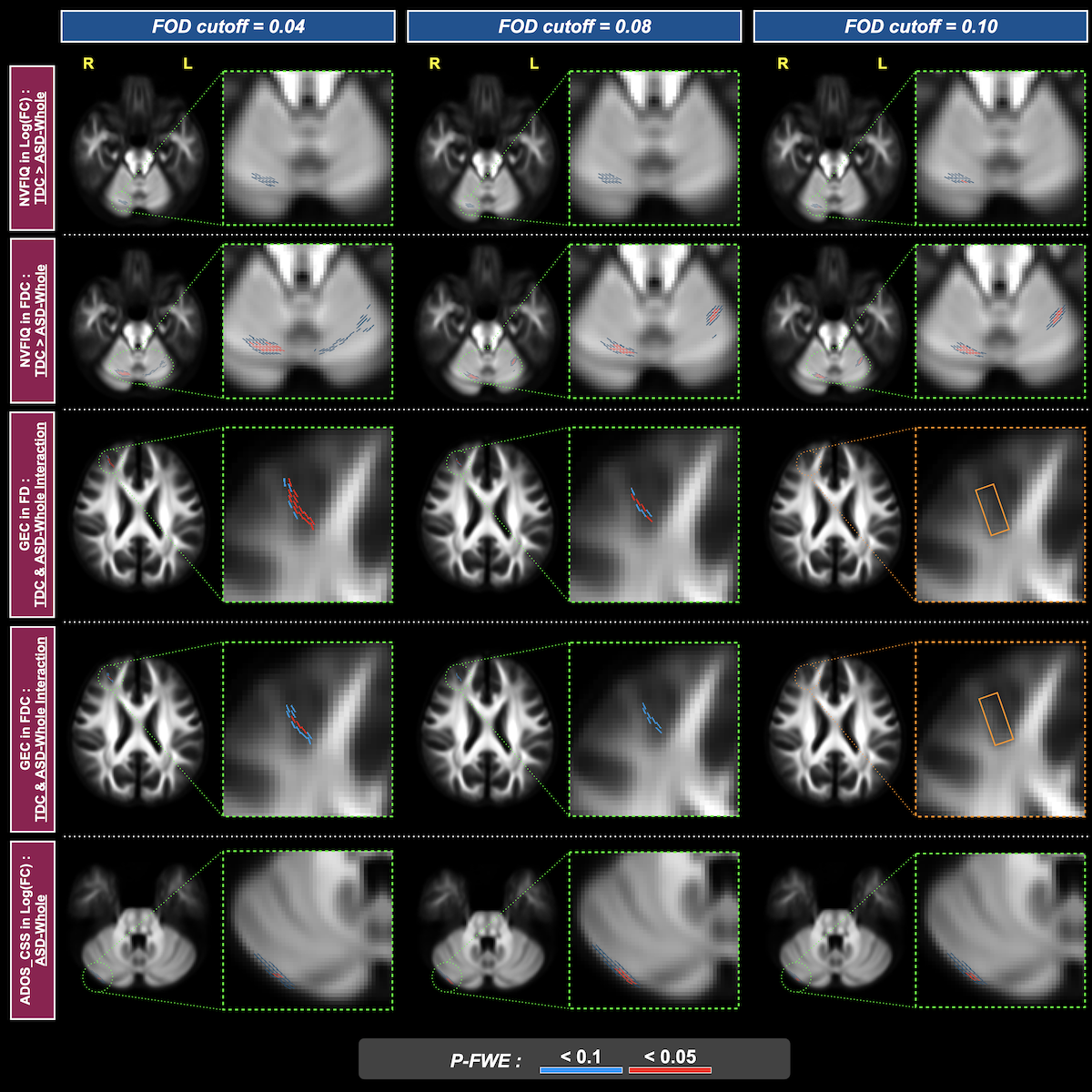


**Figure S4:** Results of dimensional brain-behavior analysis using an FOD cutoff threshold of 0.04 (left column), 0.08 (middle column), and 0.10 (right column). Each row illustrates the correlations between a fixel metric and a behavior score, in comparison to the outcomes under the FOD cutoff of 0.06 shown in **Figures 2 and 3** of the main content. The rectangle colored in orange indicates the missing significance comparing to **Figure 3**. Fixels are colored in red for *P-FWE* < 0.05; fixels colored in blue indicate *P-FWE* < 0.1 and are used to assist identification of the associated brain structure.

Interestingly, changing the FOD threshold appears to alter the FBA results oppositely in the cerebrum and cerebellum, as observed from both the categorical comparisons and dimensional analysis above. The application of a higher FOD cutoff value results in a reduced significance level in the cerebrum but an enhanced statistics in the cerebellum, either based on the FD or FDC metric. The explanation for this contrary tendency is not straightforward, as such a tendency might be dependent on the spatial location and study-specific. Ideally, one could consider using a lower FOD threshold, given sufficient quality of dMRI data. The outcomes provided in this section suggest that the default FOD cutoff of 0.06 used in this study might be the right balance for investigating both the cerebrum and cerebellum.

**E. FBA at a significance level of *P-FWE* = 0.01**

This section provides the results of using a more stringent statistical level of *P-FWE* < 0.01, with all other processing procedures and parameters identical to those in the main article. Supplementary **Figures S5-6** show the results for the categorical comparisons and dimensional analysis, respectively.


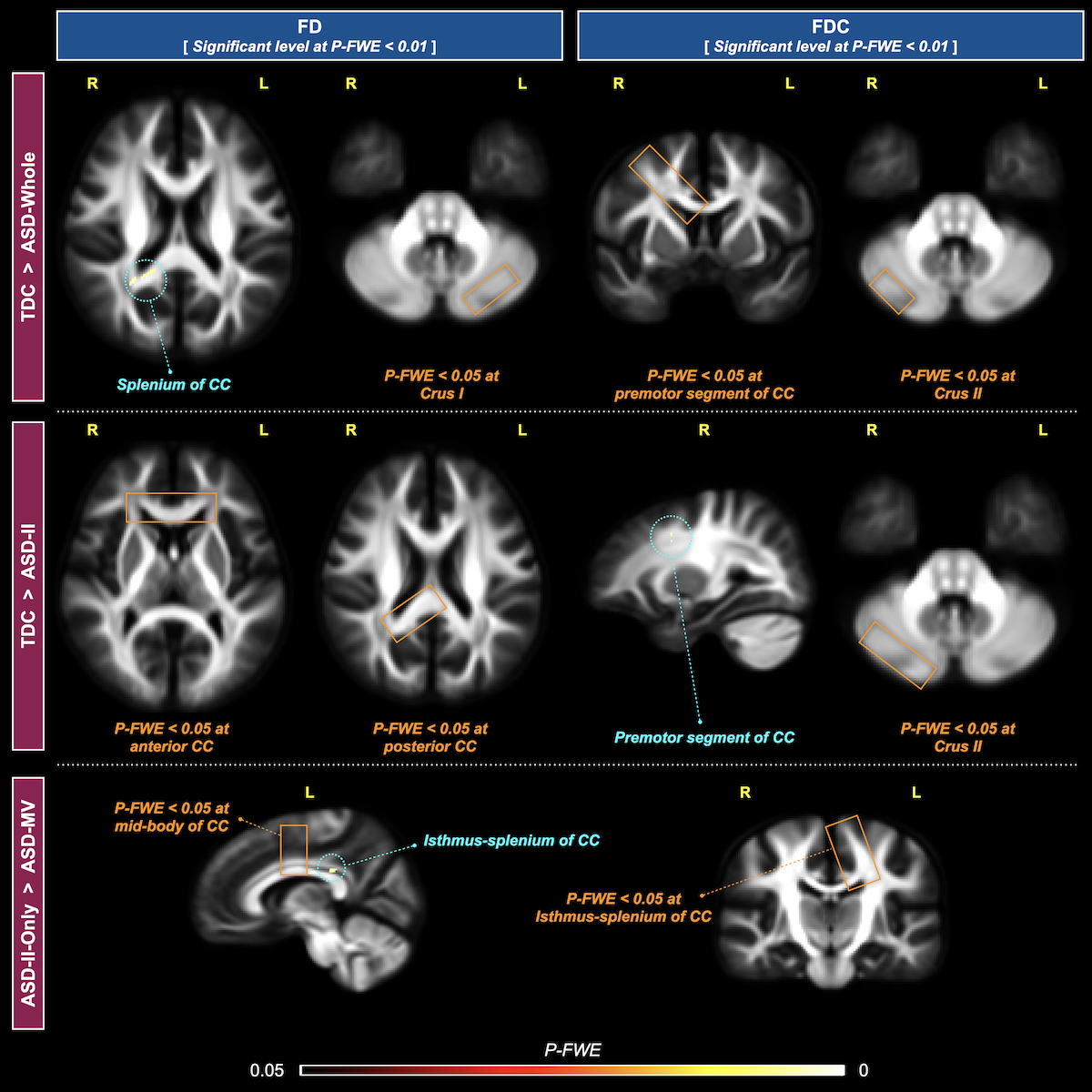


**Figure S5:** Results for categorical comparisons under a statistical threshold of *P-FWE* < 0.01. Left column – metric FD; right column – metric FDC. Upper block ⎯ TDC > ASD-Whole; middle block ⎯ TDC > ASD-II; bottom block ⎯ ASD-II-Only > ASD-MV. Fixels within blue dashed circles reached the significance level of *P-FWE* < 0.01. The rectangles colored in orange highlight brain regions where fixels reached *P-FWE* < 0.05 but did not pass the statistical testing using *P-FWE* < 0.01.


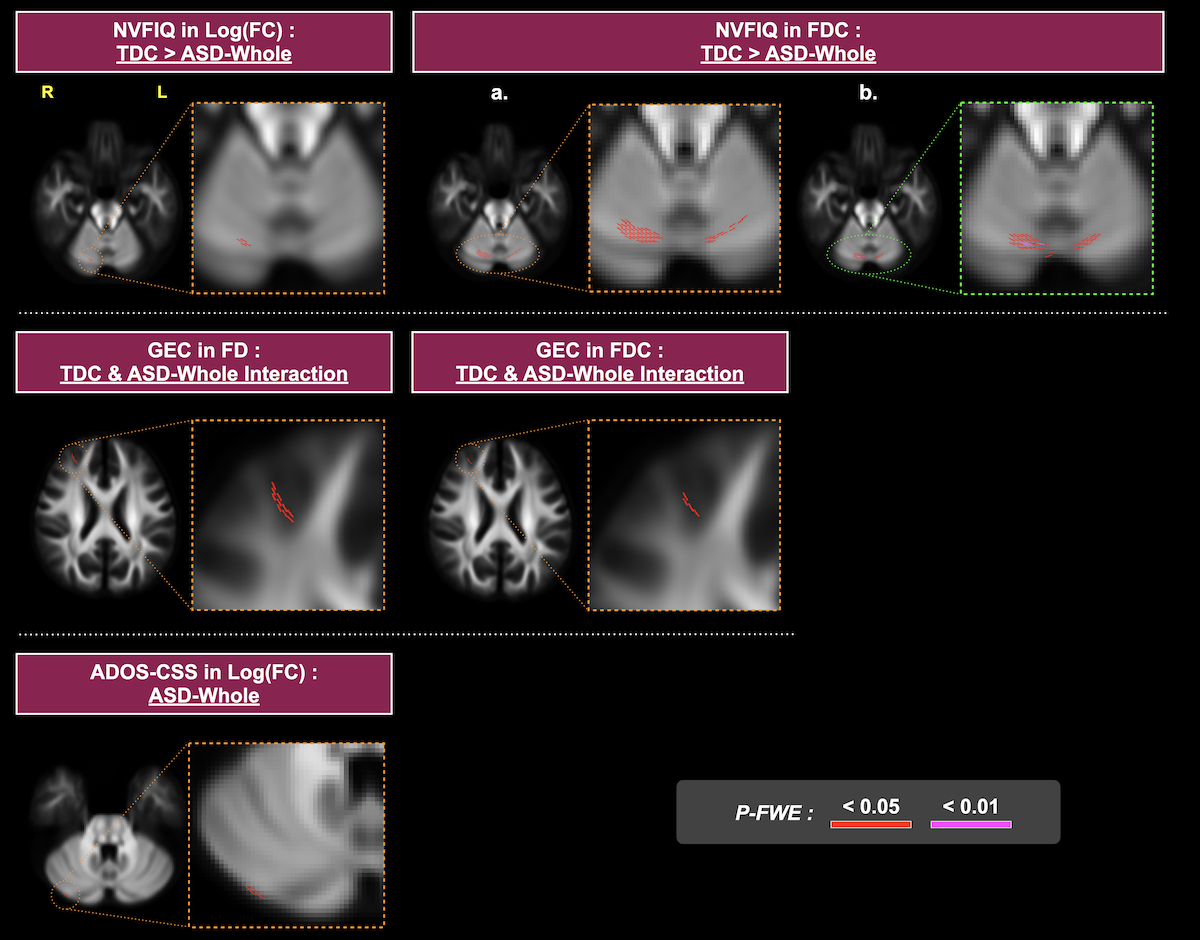


**Figure S6:** Results of dimensional analysis under a statistical threshold of *P-FWE < 0.01*. Upper row – NVFIQ: The only significance under this more restricted threshold is in the positive correlation of NVFIQ and FDC. Panel a shows the same slice in main content, where no significant results appear at *P-FWE* < 0.01. Panel b is the result at one slice below Panel a, where the significance under *P-FWE* < 0.01 are observed. Middle row – GEC: no fixels reach *P-FWE* < 0.01. Bottom row – ADOS-CSS: no fixels reach *P-FWE* < 0.01. Fixels are colored in magenta for *P-FWE* < 0.01; fixels colored in red indicate *P-FWE* < 0.05 and are used to assist identification of the associated brain structure.

**F. Voxel-based analysis based on diffusion tensor model**

This section provides the results obtained from the same study cohort using the conventional voxel-based analysis (VBA) based on the diffusion tensor metrics to benchmark against the present FBA outcomes. To this end, FA and MD maps were computed for each participant using the b=0 and b=1000 DWI volumes (90 diffusion gradient directions) extracted from the multi-shell DWI data at the preprocessed level. The FA maps of all TDC and ASD-Whole participants were used to generate a study-specific FA template. The FA and MD maps of each participant were then transformed to the template space via applying the transformation field obtained from the image registration between individual’s FA map and the template. A Gaussian kernel of 6-mm full width at half-maximum in size was applied to smooth the transformed FA and MD maps. The whole-brain statistical analysis was performed using GLM, with the threshold-free cluster enhancement applied. The nuisance variables included participant’s sex, age, medication, and relative RMS. All analyses including both the categorical and dimensional analysis as in FBA were performed for VBA. Nonparametric testing was performed using 5,000 permutations, and the significance level was defined at *P-FWE* < 0.05. All the processing steps above were done using *MRtrix3*.

Supplementary **Figure S7** shows the voxels/regions that reached statistical significance (*P-FWE* < 0.05). For the categorical comparisons, the TDC group has higher FA than the ASD-Whole at a cluster of voxels within the third ventricle. The dimensional brain-behavior analysis showed that there was a positive correlation between FA and GEC at a cluster that was also located in the brain ventricle. Both results however cannot draw a meaningful interpretation. All the results reported from FBA in the present study were not found using either FA- or MD-based VBA.


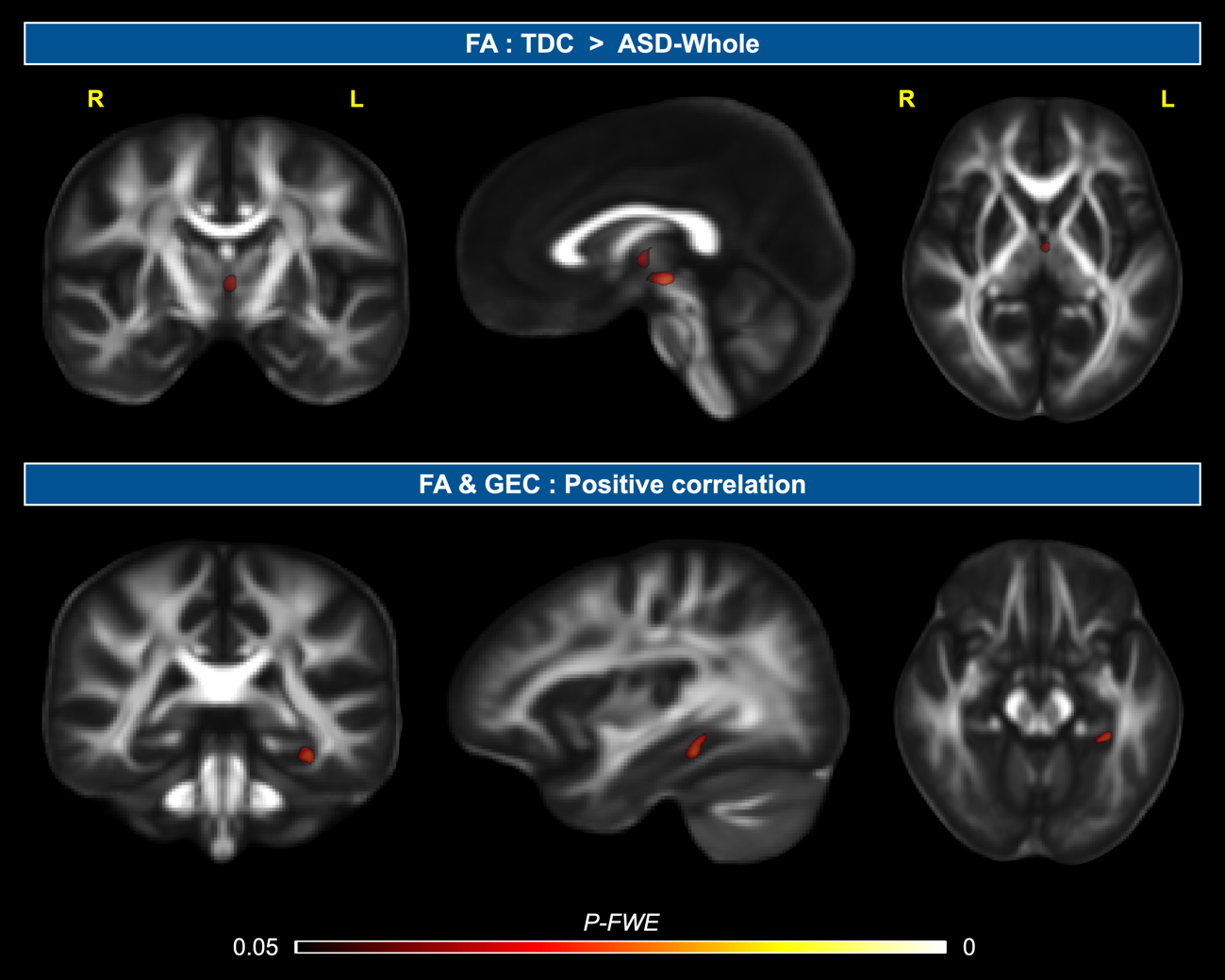


**Figure S7:** Results of VBA using the FA and MD metrics of the diffusion tensor model. Voxels that reached statistical significance (*P-FWE* < 0.05) are colored by the *P-FWE* and overlaid on the group FA template images in three orthogonal planes. Upper row ⎯ the cluster where TDC have greater FA than ASD-Whole. Bottom row ⎯ the cluster where GEC positively correlates with FA.

**Supplementary References**

1. Gabrielsen TP, Anderson JS, Stephenson KG, Beck J, King JB, Kellems R, Top DN, Jr., Russell NCC, Anderberg E, Lundwall RA, Hansen B, South M: **Functional MRI connectivity of children with autism and low verbal and cognitive performance.** *Mol Autism* 2018, **9:**67.

2. Vanderwal T, Kelly C, Eilbott J, Mayes LC, Castellanos FX: **Inscapes: A movie paradigm to improve compliance in functional magnetic resonance imaging.** *Neuroimage* 2015, **122:**222-232.
